# Chromosomal Instability May Not Be a Predictor for Immune Checkpoint Inhibitors from a Comprehensive Bioinformatics Analysis

**DOI:** 10.3390/life10110276

**Published:** 2020-11-08

**Authors:** Chiao-En Wu, Da-Wei Yeh, Yi-Ru Pan, Wen-Kuan Huang, Ming-Huang Chen, John Wen-Cheng Chang, Jen-Shi Chen, Yu-Chao Wang, Chun-Nan Yeh

**Affiliations:** 1 Division of Hematology-Oncology, Department of Internal Medicine, Chang Gung Memorial Hospital, Linkou, Chang Gung University College of Medicine, Taoyuan 333, Taiwan; 8805017@cgmh.org.tw (C.-E.W.); medfox0924@cgmh.org.tw (W.-K.H.); wen1902@cgmh.org.tw (J.W.-C.C.); js1101@cgmh.org.tw (J.-S.C.); 2 Institute of Biomedical Informatics, National Yang-Ming University, Taipei 112, Taiwan; dustin3141@gmail.com; 3 Department of General Surgery and Liver Research Center, Chang Gung Memorial Hospital, Linkou branch, Chang Gung University, Taoyuan 333, Taiwan; panyiru0331@gmail.com; 4 Department of Oncology, Taipei Veterans General Hospital, Taipei 112, Taiwan; mhchen9@vghtpe.gov.tw; 5 School of Medicine, National Yang-Ming University, Taipei 112, Taiwan; 6 Preventive Medicine Research Center, National Yang-Ming University, Taipei 112, Taiwan

**Keywords:** CIN70, chromosomal instability, immunotherapy, immune checkpoint inhibitors, tumor mutation burden, microsatellite instability

## Abstract

Immune checkpoint inhibitors (ICIs) have become the standard of care in various cancers, although their predictive tools have not yet completely developed. Here, we aimed to exam the role of 70-gene chromosomal instability signature (CIN70) in cancers, and its association with previous predictors, tumor mutation burden (TMB), and microsatellite instability (MSI), for patients undergoing ICIs, as well as the possible predictive value for ICIs. We examined the association of CIN70 with TMB and MSI, as well as the impact of these biomarkers on the survival of 33 cancer cohorts from The Cancer Genome Atlas (TCGA) databank. The predictive value of the ICIs of CIN70 in previously published reports was also validated. Using the TCGA dataset, CIN70 scores were frequently (either positively or negatively) associated with TMB, but were only significantly associated with MSI status in three types of cancer. In addition, our current study showed that all TMB, MSI, and CIN70 had their own prognostic values for survival in patients with various cancers, and that they could be cancer type-specific. In two validation cohorts (melanoma by Hugo et al. and urothelial cancer by Snyder et al.), no significant difference of CIN70 scores was found between responders and non-responders (*p*-value = 0.226 and 0.108, respectively). In addition, no overall survival difference was noted between patients with a high CIN70 and those with a low CIN70 (*p*-value = 0.106 and 0.222, respectively). In conclusion, the current study, through a comprehensive bioinformatics analysis, demonstrated a correlation between CIN70 and TMB, but CIN70 is not the predictor for cancer patients undergoing ICIs. Future prospective studies are warranted to validate these findings.

## 1. Introduction

Immune checkpoint inhibitors (ICIs) targeting immune checkpoints, such as the interaction of programmed cell death 1 (PD-1) and programmed cell death-ligand 1 (PD-L1), have been widely used in various solid cancers and have achieved impressive success in cancer treatment, leading to a new era of anticancer therapy. In 2017, pembrolizumab, an anti-PD-1 antibody, was granted the first agnostic indication by the U.S.A’s FDA for patients with microsatellite instability-high (MSI-H) or deficient mismatch repair (dMMR) solid tumors [1,2]. In 2020, pembrolizumab was approved for the treatment of tumor mutation burden-high (TMB-H) solid tumors, which are defined as ≥10 mutations/megabase (mut/Mb), as assessed using the FoundationOneCDx assay. Both MSI-H and TMB-H are associated with increased neoantigens, which elicit an immune response during ICI treatment. However, only a subset of patients has such genetic alterations and achieved durable responses following ICIs. Therefore, an exploration of the predictive biomarkers is critical in order to optimize the patients receiving benefits from ICIs.

Chromosome instability (CIN) is defined as a higher than normal rate of missegregation of whole chromosomes or fractions of chromosomes during mitosis and lacks the capacity to maintain the same chromosome number from one cell generation to the next cell generation, leading to aneuploidy (presence of extra or missing chromosomes). Not only can CIN induce aneuploidy, but aneuploidy and CIN frequently co-exist and are recognized as hallmarks of cancer [3,4]. In addition, CIN and aneuploidy form a vicious cycle, driving cancer genome chaos [5]. Aneuploidy largely translates into changes in the protein products of these genes, which can alter the balance of various protein complexes or pathways, leading to malfunctioning biological processes. CIN involves intratumor heterogenicity, cancer evaluation, host immunity, and gene mutation, which can be immunogenic, as well as increased immune evasion [6]. However, current evidence for the correlation of CIN and its response to ICIs is still unknown. Only some studies have reported that aneuploidy may influence the response to ICIs, but results are not consistent [7,8].

The association between CIN and TMB/MSI has solely been evaluated, and the predictive value of CIN in ICIs is unknown. Carter et al. developed gene expression signatures for CIN, CIN25, and CIN70, using a computational approach to identify the specific genes whose expressions were consistently correlated with the total functional aneuploidy across multiple cancer types, that could also predict patient survival and prognosis [9]. Therefore, our study aimed to evaluate the correlation of CIN70 and TMB/MSI using comprehensive bioinformatics analysis, as well as the predictive value of CIN70 in response to ICIs. This study provided additional evidence in researching novel biomarkers for immunotherapy.

## 2. Results

### 2.1. Distribution of CIN70, TMB, and MSI in 33 Cancers

**Figure 1:**
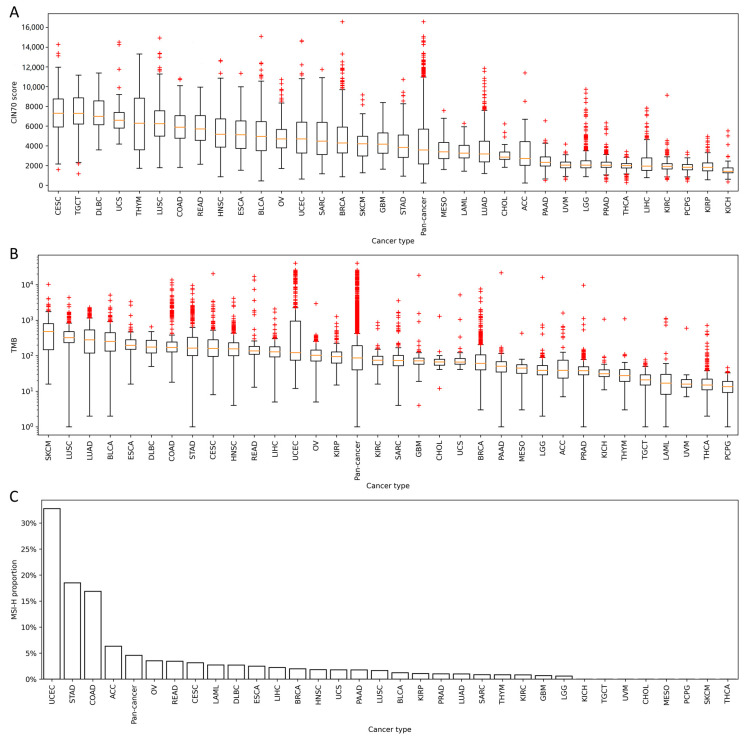
Distributions of 70-gene chromosomal instability signature (CIN70), tumor mutation burden (TMB) and microsatellite instability-high (MSI-H) in 33 cancers and pan-cancer. (**A**) The distribution of CIN70 across 33 cancers and pan-cancer. (**B**) The distribution of TMB across 33 cancers and pan-cancer. (**C**) The proportion of MSI-H samples across 33 cancers and pan-cancer.

To assess the distribution of CIN70 and TMB across various cancers, we firstly downloaded the gene expression data and somatic mutation data from The Cancer Genome Atlas (TCGA) database, in which data were collected across 33 cancer types. The CIN70 and TMB were calculated for each sample and the distribution of CIN70 and TMB in 33 cancers are shown in Figure 1A,B. In addition, the MSI status for each sample was predicted using the MSIseq tool with a high confidence [10,11]. The proportion of MSI-H samples in each cancer type is shown in Figure 1C. Of note, eight cancer types had no MSI-H samples predicted by MSIseq.

### 2.2. TMB, MSI, and CIN70 in Pan-Cancer Dataset

**Figure 2:**
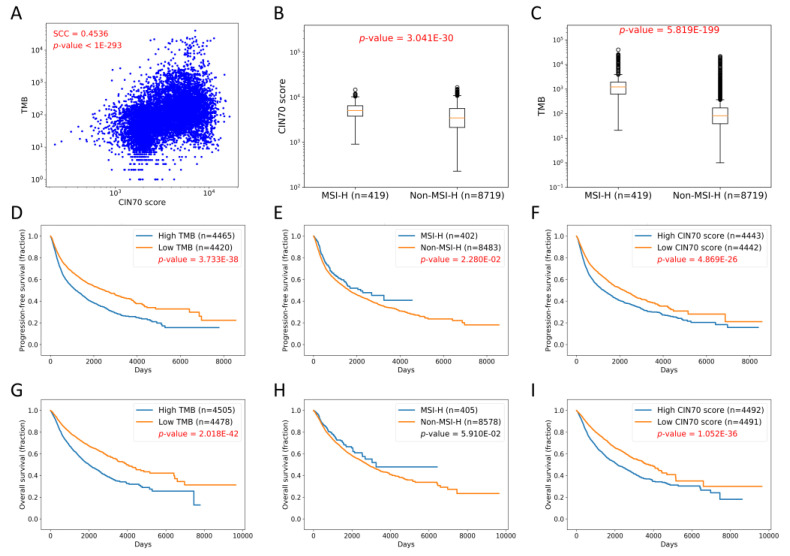
Pan-cancer data analysis. (**A**) Correlation between CIN70 and TMB (SCC = 0.4536, *p*-value < 1.0 × 10^−293^). (**B**) Association between CIN70 and MSI status (*p*-value = 3.041 × 10^−30^, Mann–Whitney U test). (**C**) Association between TMB and MSI status (*p*-value = 5.819 × 10^−199^, Mann–Whitney U test). (**D**) Association between TMB and progression-free survival (PFS) (*p*-value = 3.733 × 10^−38^, log-rank test). (**E**) Association between MSI status and PFS (*p*-value = 0.0228, log-rank test). (**F**) Association between CIN70 and PFS (*p*-value = 4.869 × 10^−26^, log-rank test). (**G**) Association between TMB and overall survival (OS) (*p*-value = 2.018 × 10^−42^, log-rank test). (**H**) Association between MSI status and OS (*p*-value = 0.0591, log-rank test). (**I**) Association between CIN70 and OS (*p*-value = 1.052 × 10^−36^, log-rank test).

All of the cancer types were pooled together as the pan-cancer dataset for the analysis. The correlation between CIN70 and TMB from the TCGA database was evaluated using the Spearman correlation coefficient (SCC). CIN70 was significantly corelated with TMB (Figure 2A). Both CIN70 and TMB were significantly associated with MSI status (Figure 2B,C). TMB-H and a high CIN70 were associated with a poor progression-free survival (PFS) and overall survival (OS); in contrast, MSI-H was marginally associated with a favorable PFS, but not OS (Figure 2D–I).

### 2.3. Correlation between CIN70 and TMB in 33 Cancer Types

**Figure 3:**
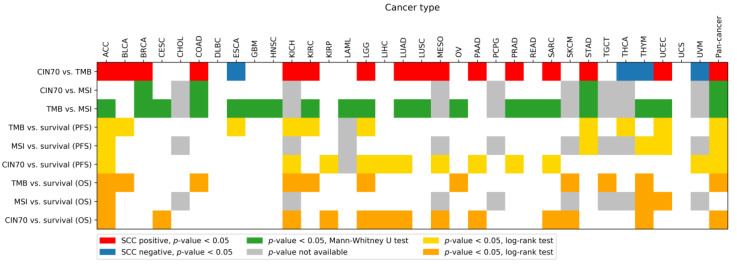
Association between CIN70, TMB, MSI status and survival in 33 cancer types and pan-cancer.

We further examined the correlation between CIN70 and TMB in 33 cancers from the TCGA database using the SCC method. The association between CIN70 and TMB was found to be significant in 19 out of 33 cancer types using SCC. Of note, not all of the associations were positively correlated, and there were some cancer types in which CIN70 and TMB were negatively correlated (esophageal carcinoma (ESCA), thyroid carcinoma (THCA), thymoma (THYM), and uveal melanoma (UVM); Figure 3, Supplementary Figure S1 in the Supplementary Materials).

### 2.4. Association between CIN70 and MSI Status in 25 Cancer Types with MSI-H Samples

The CIN70 scores among the MSI-H and non-MSI-H samples were evaluated for each cancer type. Among the 25 cancer types with MSI-H samples, the CIN70 scores were significantly higher in the MSI-H group than non-MSI-H group within only three cancer types (breast invasive carcinoma (BRCA), colon adenocarcinoma (COAD), and stomach adenocarcinoma (STAD); Figure 3, Supplementary Figure S2).

### 2.5. Association between TMB and MSI Status in 25 Cancer Types with MSI-H Samples

In 25 cancer types harboring MSI-H samples from the TCGA databank, MSI-H was significantly associated with a higher TMB in 19 cancer types, indicating that the samples with MSI-H tend to have a higher TMB in most cancers (Figure 3, Supplementary Figure S3).

### 2.6. Association between TMB, MSI, CIN70, and PFS

Regarding PFS, TMB is significantly associated with a PFS difference in 9 out of 32 cancer types (Figure 3, Supplementary Figure S4; Supplementary Table S1). In most cancer types, patients with TMB-H had a significantly shorter PFS than patients without TMB-H, indicating TMB-H is an unfavorable prognostic factor in such cancers. However, patients with TMB-H had a significantly longer PFS than the patients without TMB-H in three cancer types (bladder urothelial carcinoma (BLCA), STAD, and uterine corpus endometrial carcinoma (UCEC); Supplementary Figure S4).

MSI status was significantly associated with PFS in four cancer types (Figure 3, Supplementary Figure S5; Supplementary Table S2). In adrenocortical carcinoma (ACC) and THYM, non-MSI-H patients had a significantly longer PFS than MSI-H patients. However, contrary results were found in STAD and UCEC (Supplementary Figure S5).

Using the median of CIN70, we performed the survival analysis, comparing the high CIN70 and low CIN70 patients in each cancer type. CIN70 was significantly associated with PFS in 11 of the 32 cancer types (Figure 3, Supplementary Figure S6; Supplementary Table S3). In all 11 cancer types, a high CIN70 was associated with a poor PFS (Supplementary Figure S6).

### 2.7. Association between TMB, MSI, and CIN70 and OS

Regarding OS, TMB was significantly associated with an OS difference in 10 out of 33 cancer types (Figure 3, Supplementary Figure S7; Supplementary Table S1). Specifically, the patients with TMB-H had a shorter OS than those without TMB-H in six cancers. On the other hand, patients with TMB-H had a longer OS than those without TMB-H in four cancers (BLCA, ovarian serous cystadenocarcinoma (OV), skin cutaneous melanoma (SKCM), and testicular germ cell tumors (TGCT); Supplementary Figure S7).

In 25 cancer types with MSI-H samples, MSI status was significantly associated with OS in three cancer types (Figure 3, Supplementary Figure S8; Supplementary Table S2). In ACC and THYM, non-MSI-H patients had a significantly longer OS than the MSI-H patients. However, MSI-H patients had a longer OS than the non-MSI-H patients in UCEC (Supplementary Figure S8).

The results showed that in 12 out of the 33 cancer types, CIN70 was significantly associated with OS (Figure 3, Supplementary Figure S9; Supplementary Table S3). In most of the cancer types, patients with a high CIN70 had a shorter OS than those with a low CIN70. However, unlike the prognostic value of CIN70, PFS showing CIN70 is a universally poor prognostic factor, and patients with a high CIN70 had a favorable OS in two cancer types (cervical squamous cell carcinoma and endocervical adenocarcinoma (CESC) and THYM; Supplementary Figure S9).

### 2.8. Validation of CIN70 in other Cohort Regarding ICI Response 

**Figure 4:**
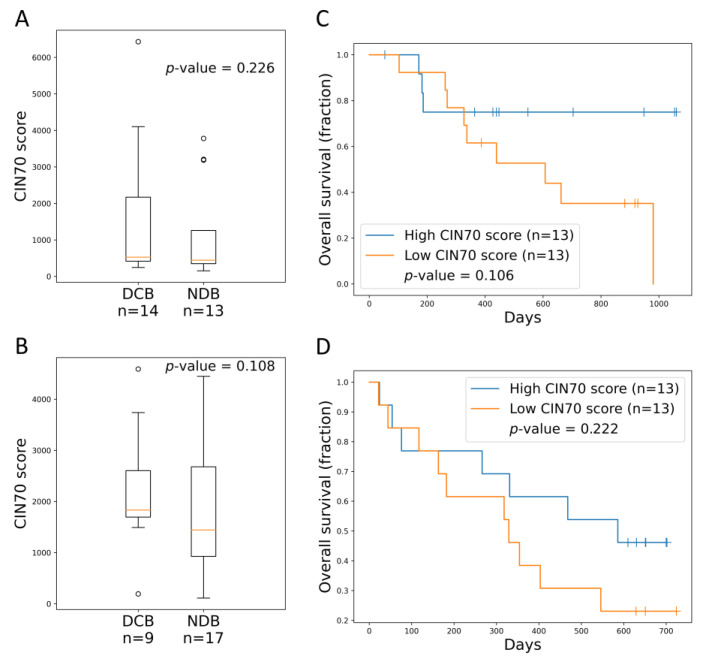
Association between CIN70 and immune checkpoint inhibitor responses. (**A**) CIN70 among responders and non-responders in the Hugo et al. melanoma cohort (*p*-value = 0.226, Mann–Whitney U test). (**B**) CIN70 among responders and non-responders in the Snyder et al. urothelial cancer cohort (*p*-value = 0.108, Mann-Whitney U test). (**C**) Survival analysis comparing OS of patients with high/low CIN70 score in the Hugo et al. melanoma cohort (*p*-value = 0.106, log-rank test). (**D**) Survival analysis comparing OS of patients with high/low CIN70 score in the Snyder et al. urothelial cancer cohort (*p*-value = 0.222, log-rank test). DCB: durable clinical benefit, NDB: no durable benefit.

Finally, two validation cohorts by Hugo et al. [12] and Snyder et al. [13] were assessed for the association between CIN70 and treatment outcomes in melanoma and urothelial cancer patients treated with ICIs. Regarding the CIN70 scores between responders and non-responders, no significant difference was found (*p*-value = 0.226 and 0.108, respectively; Figure 4A,B). The OS was further evaluated and no survival difference was noted between patients with a high CIN70 and those with a low CIN70 (*p*-value = 0.106 and 0.222, respectively; Figure 4C,D). These findings suggest CIN70 is not a prognostic or predictive factor in melanoma and urothelial cancer patients undergoing ICIs.

## 3. Discussion

In the current study, using the TCGA dataset, CIN70 was found to be associated with TMB but not MSI. In addition, it showed that all TMB, MSI, and CIN70 had their prognostic values for survival in patients with various cancers, and this may be cancer type-specific (Figure 2 and Figure 3). However, no association between CIN70 or a response to ICIs was found in the two validation cohorts (Figure 4). Further prospective studies should be performed to validate these findings.

Although the biological importance of CIN in cancer has been recognized, the molecular basis of CIN in cancers remains unclear, as CIN results from a heterogeneous mechanism. Multiple genetic alterations contribute to CIN, such as genes involving DNA damage and repair [14], mitotic checkpoint [15], chromosome condensation and segregation from mutational inactivation of *STAG2* [16,17], and possibly sister chromatid cohesion (*hSecurin*) [17,18]. Some studies have shown conflicting results [17,18], so it is challenging to unify the possible mechanisms into one general mechanism so as to explain CIN in cancers. CIN involves DNA damage and repair genes, which also were associated with TMB [19,20,21], and this can explain why CIN70 was associated with TMB in the current study (Figure 2 and Figure 3, and Supplementary Figure S1). However, the exact causality and mechanism between CIN and TMB is unclear, and could be plausibly explained by the joint result from common genetic alterations. In contrast, MSI-H mainly results from, but is not limited to, alterations of MMR genes (MLH1, MSH2, MSH6, and PMS2) [22], which do not involve the mechanism of CIN, so a limited association between CIN70 and MSI was found in the current study (Figure 3 and Supplementary Figure S2).

All of the TMB, MSI, and CIN70 had their prognostic values for survival in patients with various cancers. However, the prognostic values may be cancer type-specific (Figure 2 and Figure 3). For example, MSI-H is a poor prognostic factor for PFS and OS for ACC and THYM, but is a favorable prognostic factor for PFS and OS in UCEC. TMB-H is a good prognostic factor for both PFS and OS in BLCA, but not in other cancers. As shown by the heatmap in Figure 3, the prognostic values of these biomarkers may be limited in certain cancers (such as ACC, kidney chromophobe (KICH), and brain lower grade glioma (LGG)), but not in other cancers (BRCA, lymphoid neoplasm diffuse large B-cell lymphoma (DLBC), glioblastoma multiforme (GBM), head and neck squamous cell carcinoma (HNSC), lung squamous cell carcinoma (LUSC), rectum adenocarcinoma (READ), and uterine carcinosarcoma (UCS)). Future studies for specific cancers may be required for future clinical validation.

CIN70 could not predict the tumor response to ICIs or survival in two validation cohorts (Figure 4). An exome analysis of nivolumab in 77 lung cancer patients showed that aneuploidy was not associated with PFS, but was significantly associated with a reduced OS [7]. This finding indicates that aneuploidy is a poor prognostic factor for OS, but may not impact the activity of nivolumab as there is no change in PFS. Another study reported that tumor aneuploidy, which was assessed by somatic copy number alteration (SCNA) level based on SNP-array-based data, is correlated with markers of immune evasion and with reduced survivals of melanoma patients undergoing ICIs [8]. Both of the previous studies reflected the nature that aneuploidy is a poor prognostic factor in patients undergoing ICIs, but no predictive role for ICI response was noted. However, the two validation studies could not confirm the same finding that aneuploidy assessed by CIN70 is a prognostic marker in patients undergoing ICIs [12,13]. The major difference may be that we used different biomarkers to assess aneuploidy in different clinical cohorts. Therefore, future studies are warranted to validate these findings and provide additional scientific understandings about aneuploidy in ICI response.

As a retrospective study, limitations exist in the current study. All of the preliminary data were assessed from the TCGA databank, and in some cases, specific cancer types were limited, resulting in possibly unreliable results. No details of the specific treatment were available from such a databank. In addition, various correlations between TMB/MSI and CIN70 were noted, so the correlation may be cancer-type specific. Furthermore, only the two validation cohorts were assessed, and they were limited to melanoma and urothelial cancer patients, so the findings may not be generalized to all cancer types. This resulted from the limited efficacy of ICIs in cancers other than melanoma if unselective patients were enrolled without adequate biomarkers to enrich the responders.

In conclusion, the current study using comprehensive bioinformatics analysis demonstrated the correlation between CIN and TMB, but CIN70 was not a predictor for cancer patients undergoing ICIs. In addition, CIN70 may serve as a prognostic factor in various cancers. Future prospective studies are warranted to validate these findings.

## 4. Materials and Methods

### 4.1. Datasets

The RNA-seq gene expression data and somatic mutation data for 33 cancer types were curated from the TCGA database. Specifically, somatic mutation data were accessed from the TCGA multi-center mutation calling in multiple cancers (MC3) project, in which seven variant calling methods were applied to detect mutations [23]. The clinical information of the TCGA dataset was retrieved from the TCGA-clinical data resource (TCGA-CDR) outcome [24], which includes OS and PFS information (there is no PFS information for acute myeloid leukemia (LAML)). The samples with primary solid tumors or primary blood-derived cancers were screened for comprehensive analysis. For the investigation of the association between the CIN70 and ICI responses, the gene expression data and clinical information were obtained from Hugo et al. [12] and Snyder et al. [13].

### 4.2. Determination of CIN70, TMB, and MSI Status for Each Sample

In this study, the samples with primary tumors were screened for further analysis. For determination of the CIN70 score, the gene expression read counts obtained from TCGA were transformed into transcript per million (TPM) values, and the CIN70 score for each sample was calculated as the sum of the expression of 70 chromosomal instability genes identified by Carter et al. [9]. TMB was defined as the total number of mutations in a sample. Therefore, TMB was determined by counting the number of mutations in the TCGA MC3 data. Furthermore, with the somatic mutation data obtained from TCGA, the MSI status (MSI-H or non-MSI-H) was predicted using the MSIseq tool with a high confidence [10,11].

### 4.3. Statistical Analysis

For each cancer type, the Spearman correlation coefficient (SCC) was calculated to indicate the correlation between CIN70 and TMB. In addition, the Mann-Whitney U test was used to test whether MSI-H samples had a higher CIN70/TMB. For the survival analysis, the median value of CIN70/TMB was used for the classification. The Kaplan-Meier survival curves were plotted, comparing patients with a high and low CIN70/TMB. The log-rank test was used to determine the significance. A *p*-value less than 0.05 was considered significant for all of the statistical analyses. 

## Data Availability

The datasets used and/or analyzed during the current study are available from the corresponding author on reasonable request.

## Supplementary Materials

The following are available online at https://www.mdpi.com/2075-1729/10/11/276/s1, Figure S1: Correlation between CIN70 and TMB in 33 cancer types, Figure S2: Association between CIN70 and MSI status in 25 cancer types with MSI-H samples, Figure S3: Association between TMB and MSI status in 25 cancer types with MSI-H samples, Figure S4: Association between TMB and progression-free survival, Figure S5: Association between MSI status and progression-free survival, Figure S6: Association between CIN70 and progression-free survival, Figure S7: Association between TMB and overall survival, Figure S8: Association between MSI status and overall survival, Figure S9: Association between CIN70 and overall survival, Table S1: Association between TMB and OS/PFS, Table S2: Association between MSI and OS/PFS, Table S3: Association between CIN70 and OS/PFS.

## Abbreviations

ACC	Adrenocortical carcinoma
BLCA	Bladder urothelial carcinoma
BRCA	Breast invasive carcinoma
CESC	Cervical squamous cell carcinoma and endocervical adenocarcinoma
CHOL	Cholangiocarcinoma
COAD	Colon adenocarcinoma
DLBC	Lymphoid neoplasm diffuse large B-cell lymphoma
ESCA	Esophageal carcinoma
GBM	Glioblastoma multiforme
HNSC	Head and neck squamous cell carcinoma
KICH	Kidney chromophobe
KIRC	Kidney renal clear cell carcinoma
KIRP	Kidney renal papillary cell carcinoma
LAML	Acute myeloid leukemia
LGG	Brain lower grade glioma
LIHC	Liver hepatocellular carcinoma
LUAD	Lung adenocarcinoma
LUSC	Lung squamous cell carcinoma
MESO	Mesothelioma
OV	Ovarian serous cystadenocarcinoma
PAAD	Pancreatic adenocarcinoma
PCPG	Pheochromocytoma and paraganglioma
PRAD	Prostate adenocarcinoma
READ	Rectum adenocarcinoma
SARC	Sarcoma
SKCM	Skin cutaneous melanoma
STAD	Stomach adenocarcinoma
TGCT	Testicular germ cell tumors
THCA	Thyroid carcinoma
THYM	Thymoma
UCEC	Uterine corpus endometrial carcinoma
UCS	Uterine carcinosarcoma
UVM	Uveal melanoma
