# Supplementary material for: Chromosomal Instability May Not Be a Predictor for Immune Checkpoint Inhibitors from a Comprehensive Bioinformatics Analysis
Source: Life (Basel). 2020 Nov 8;10(11):276. doi: 10.3390/life10110276 (PMC7695172; doi:10.3390/life10110276)
Supplement: Supplementary file 1 [file life-10-00276-s001.zip › Supplementary Figures.pdf]

Supplementary Figures

Supplementary Figure S1. Correlation between CIN70 and TMB in 33 cancer types.
Supplementary Figure S2. Association between CIN70 and MSI status in 25 cancer types with MSI-H samples.
Supplementary Figure S3. Association between TMB and MSI status in 25 cancer types with MSI-H samples.
Supplementary Figure S4. Association between TMB and progression-free survival.
Supplementary Figure S5. Association between MSI status and progression-free survival.
Supplementary Figure S6. Association between CIN70 and progression-free survival.
Supplementary Figure S7. Association between TMB and overall survival.
Supplementary Figure S8. Association between MSI status and overall survival.
Supplementary Figure S9. Association between CIN70 and overall survival.

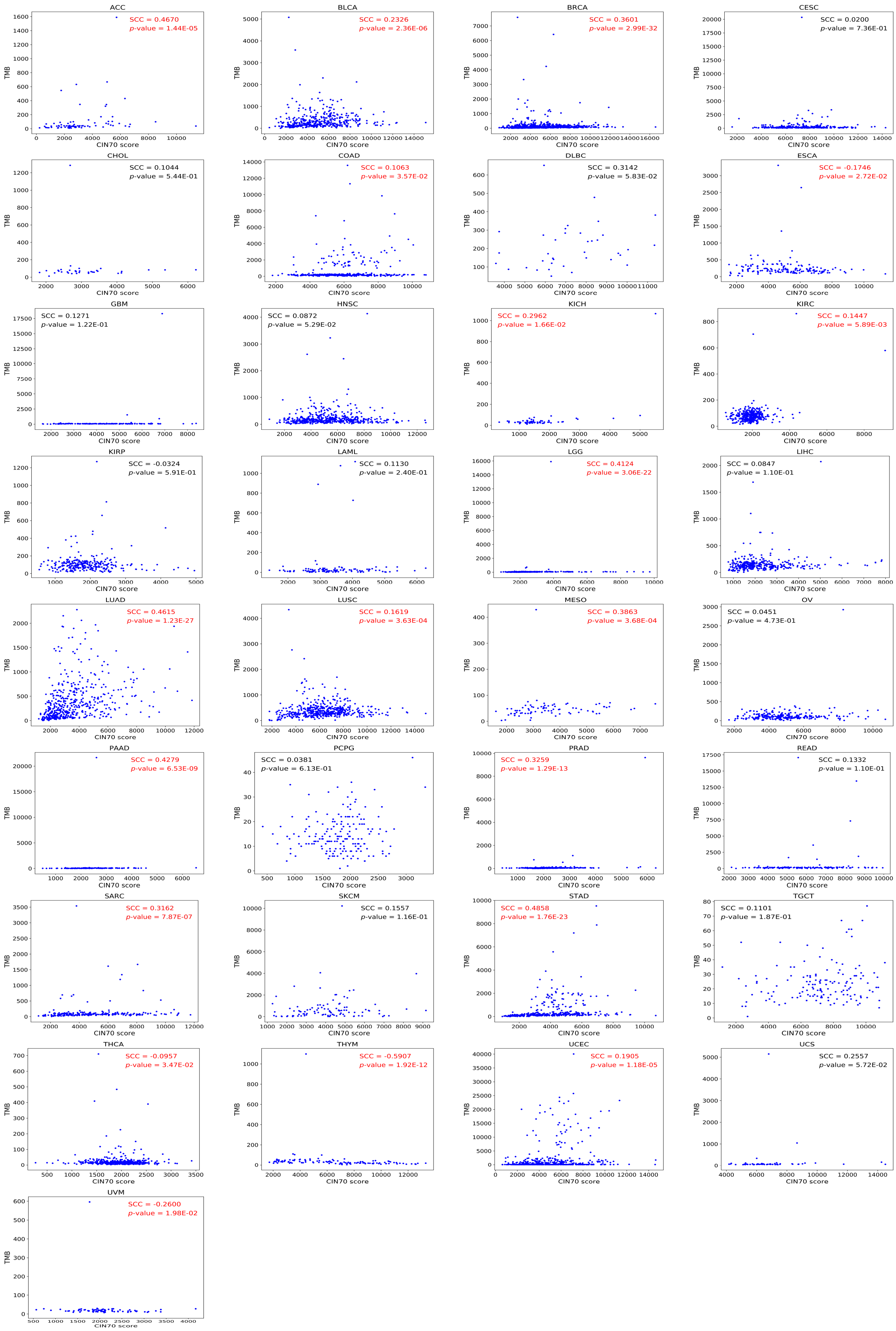

Supplementary Figure S1. Correlation between CIN70 and TMB in 33 cancer types. Spearman correlation coefficient (SCC) and the corresponding *p*-value are calculated for each cancer type.

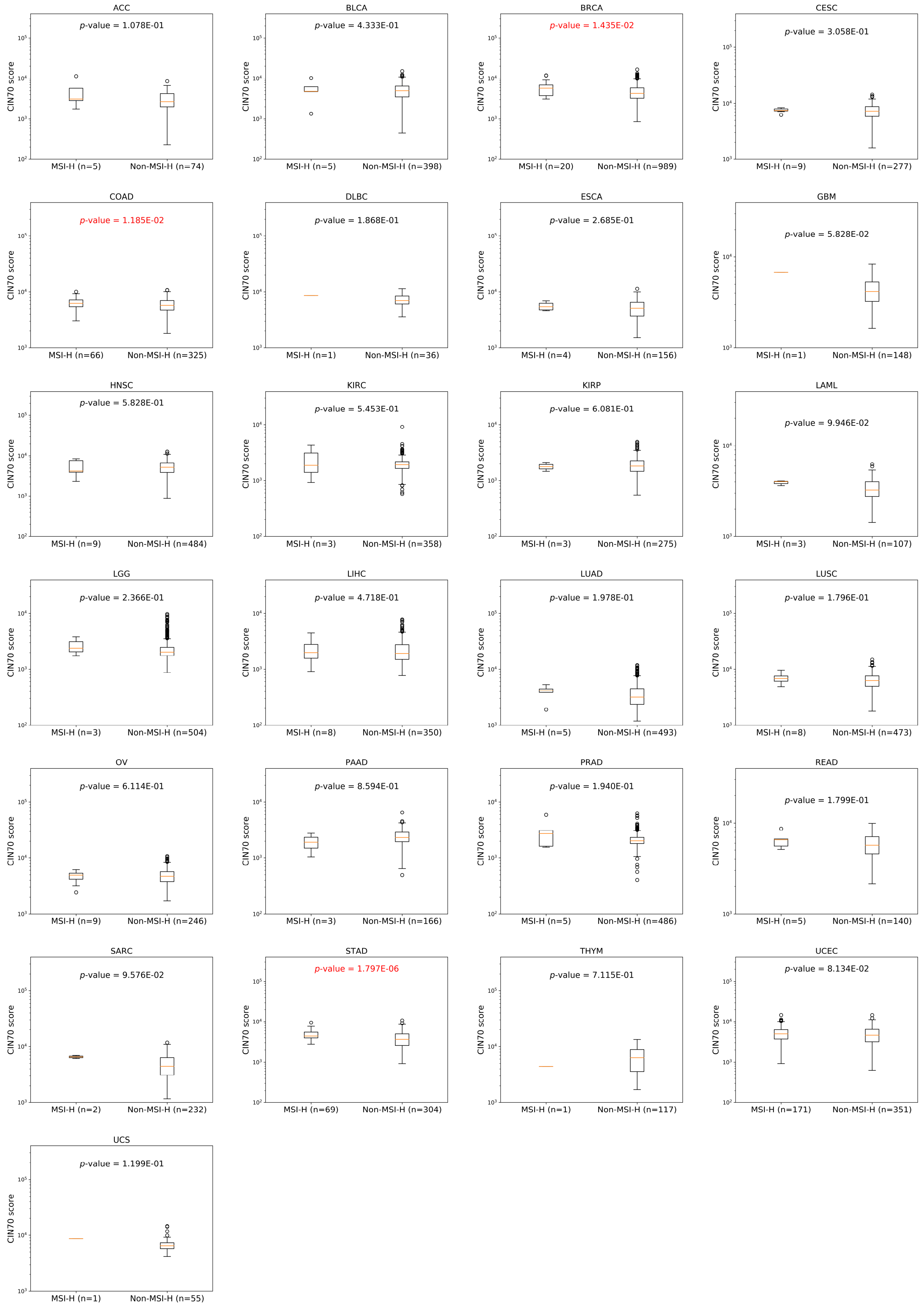

Supplementary Figure S2. Association between CIN70 and MSI status in 25 cancer types with MSI-H samples. *p*-values are given based on Mann-Whitney U test (one-sided).

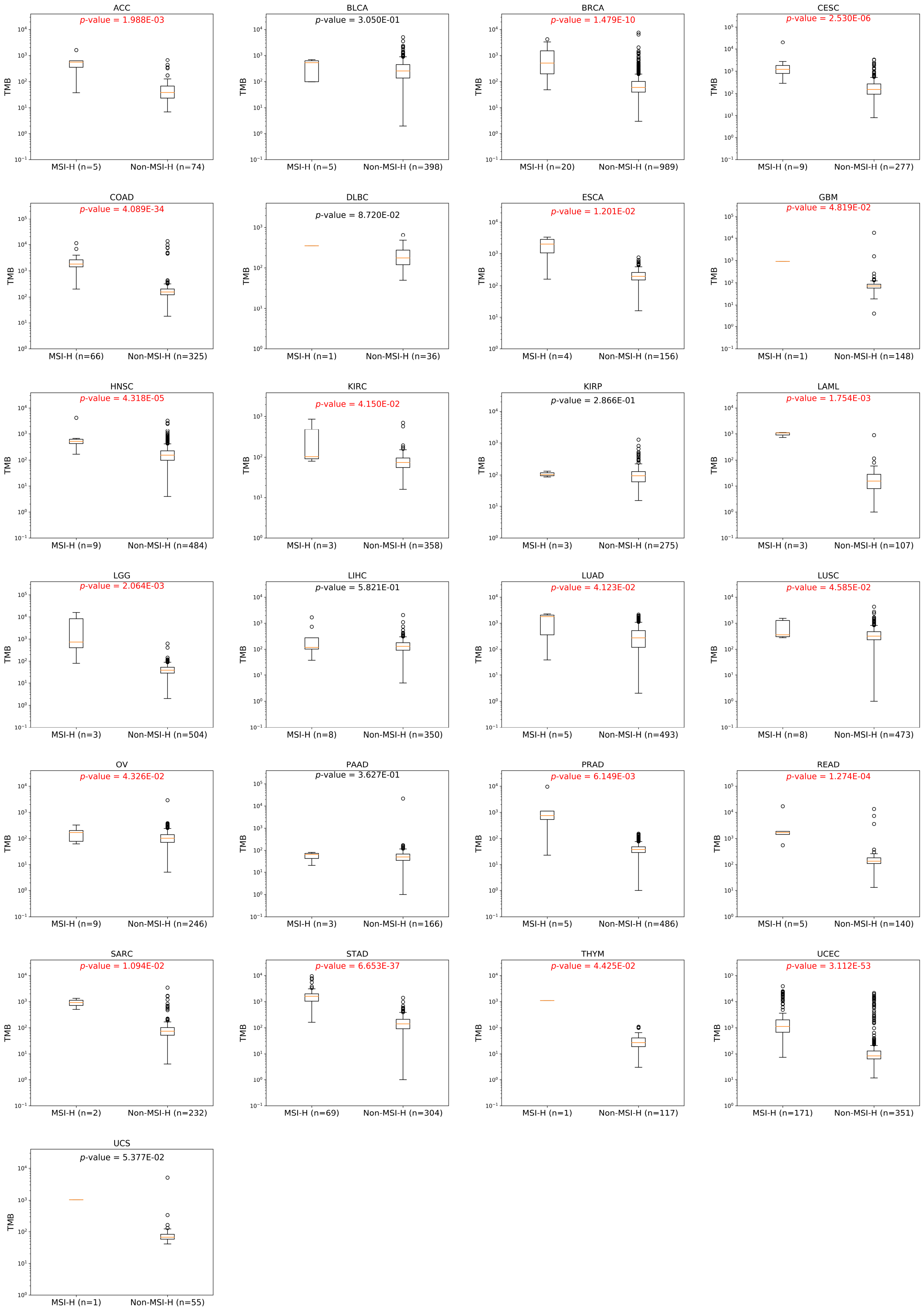

Supplementary Figure S3. Association between TMB and MSI status in 25 cancer types with MSI-H samples. *p*-values are given based on Mann-Whitney U test (one-sided).

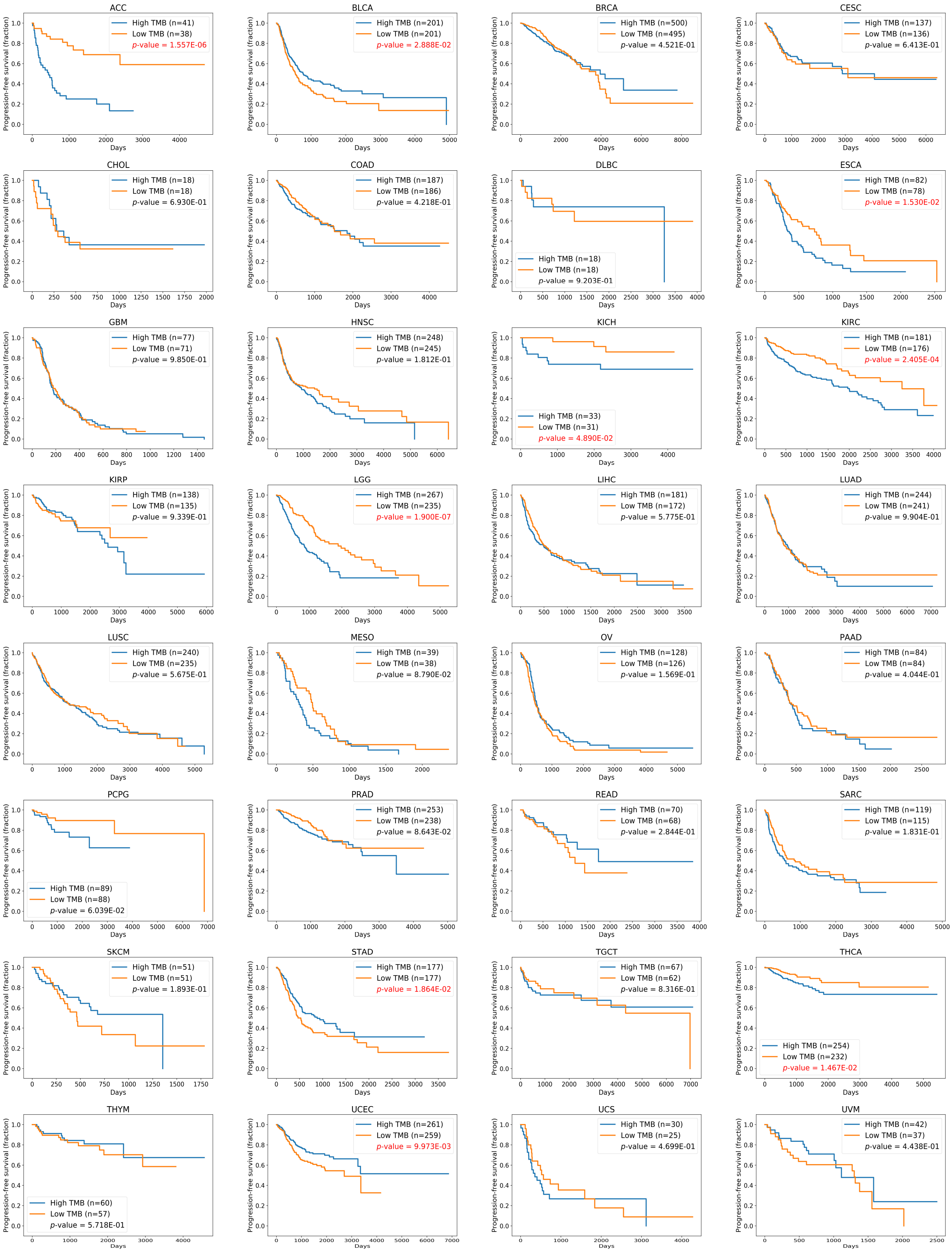

Supplementary Figure S4. Association between TMB and progression-free survival. High/low TMB groups are classified according to the median of TMB in each cancer type. *p*-values are given based on log-rank test. There is no progress-free survival information for LAML in TCGA-CDR data, resulting in 32 panels in this figure.

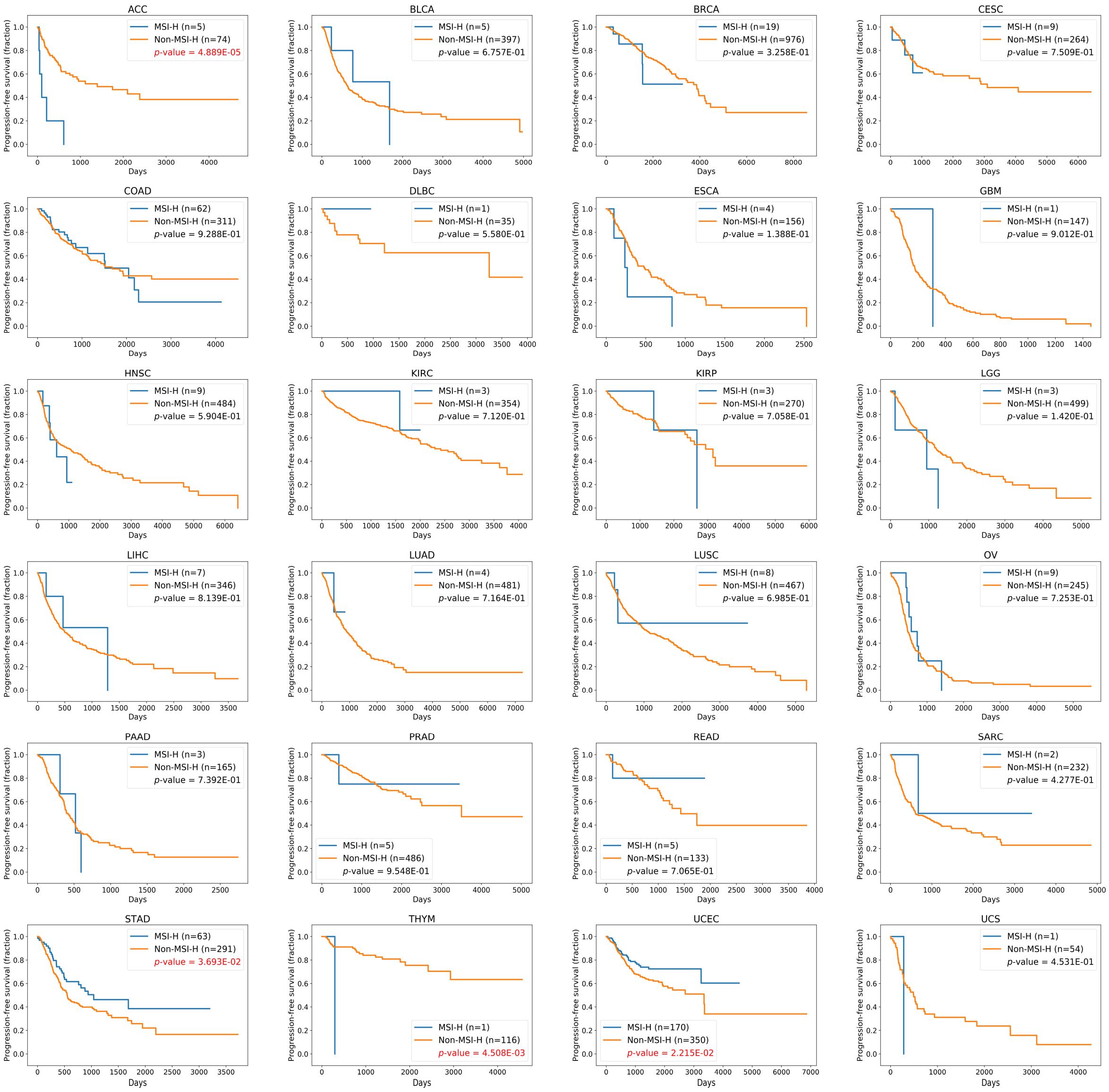

Supplementary Figure S5. Association between MSI status and progression-free survival. *p*-values are given based on log-rank test. There is no progress-free survival information for LAML in TCGA-CDR data, resulting in 24 panels in this figure.

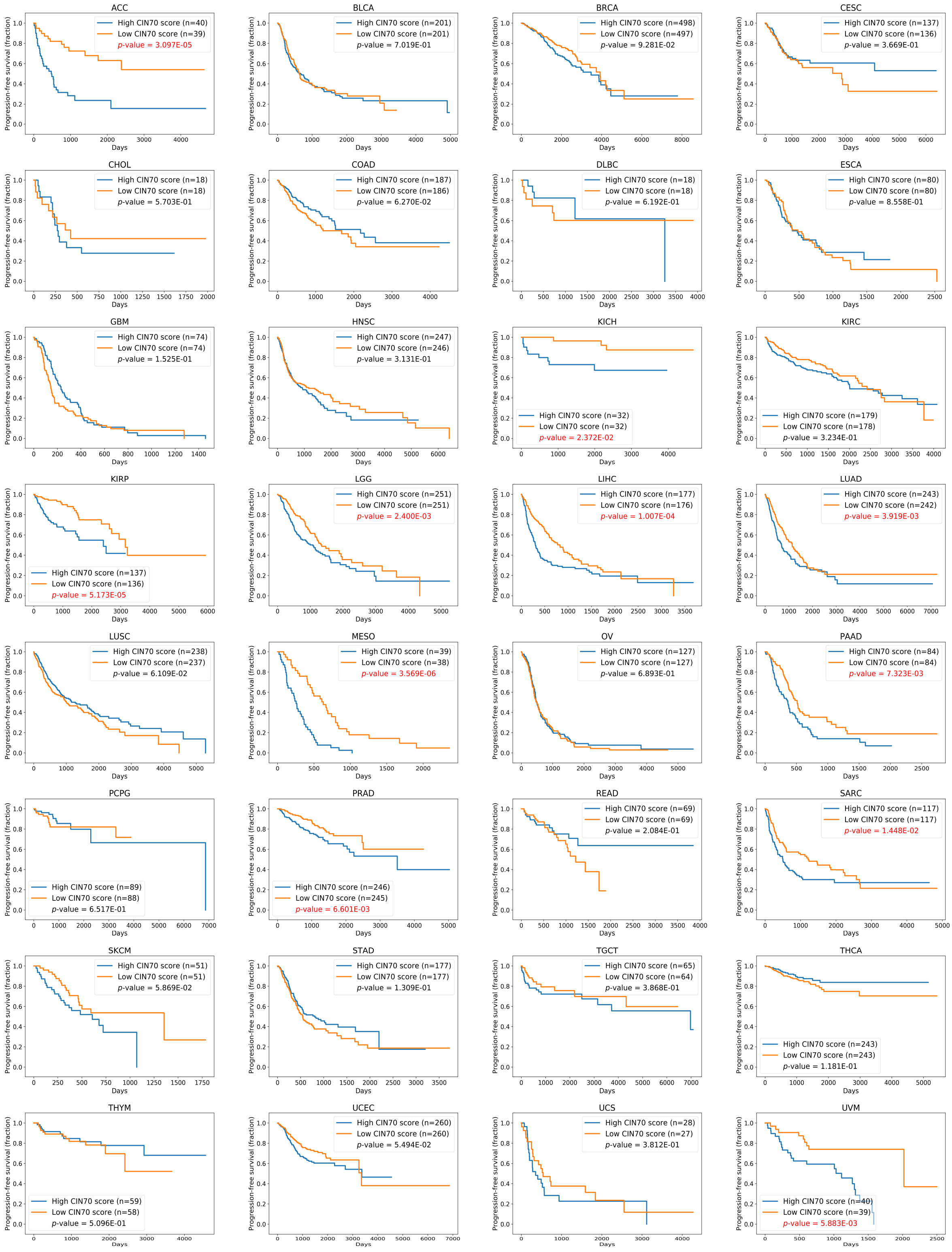

Supplementary Figure S6. Association between CIN70 and progression-free survival. High/low CIN70 groups are classified according to the median of CIN70 in each cancer type. *p*-values are given based on log-rank test. There is no progress-free survival information for LAML in TCGA-CDR data, resulting in 32 panels in this figure.

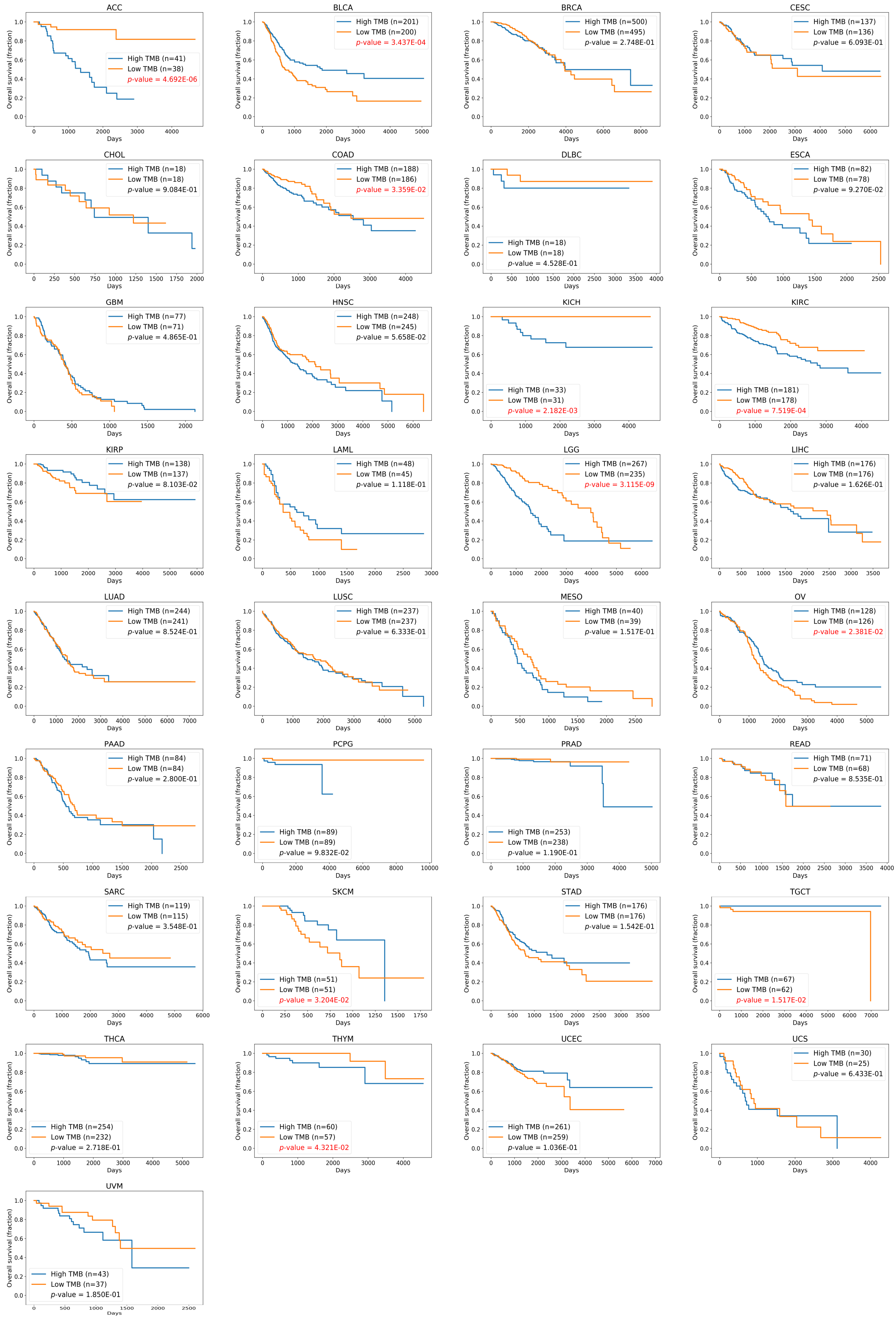

Supplementary Figure S7. Association between TMB and overall survival. High/low TMB groups are classified according to the median of TMB in each cancer type. *p*-values are given based on log-rank test.

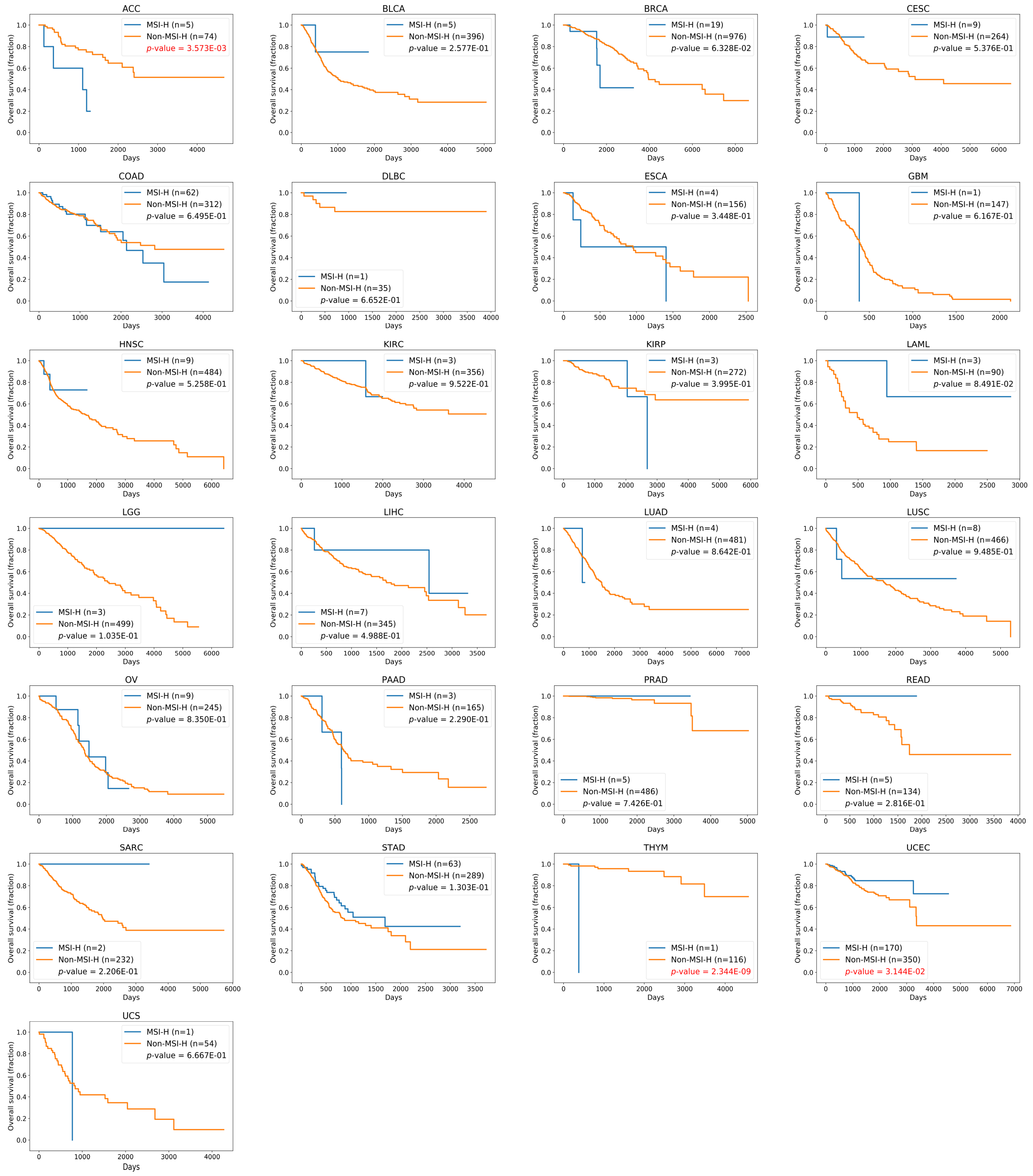

Supplementary Figure S8. Association between MSI status and overall survival. *p*-values are given based on log-rank test.

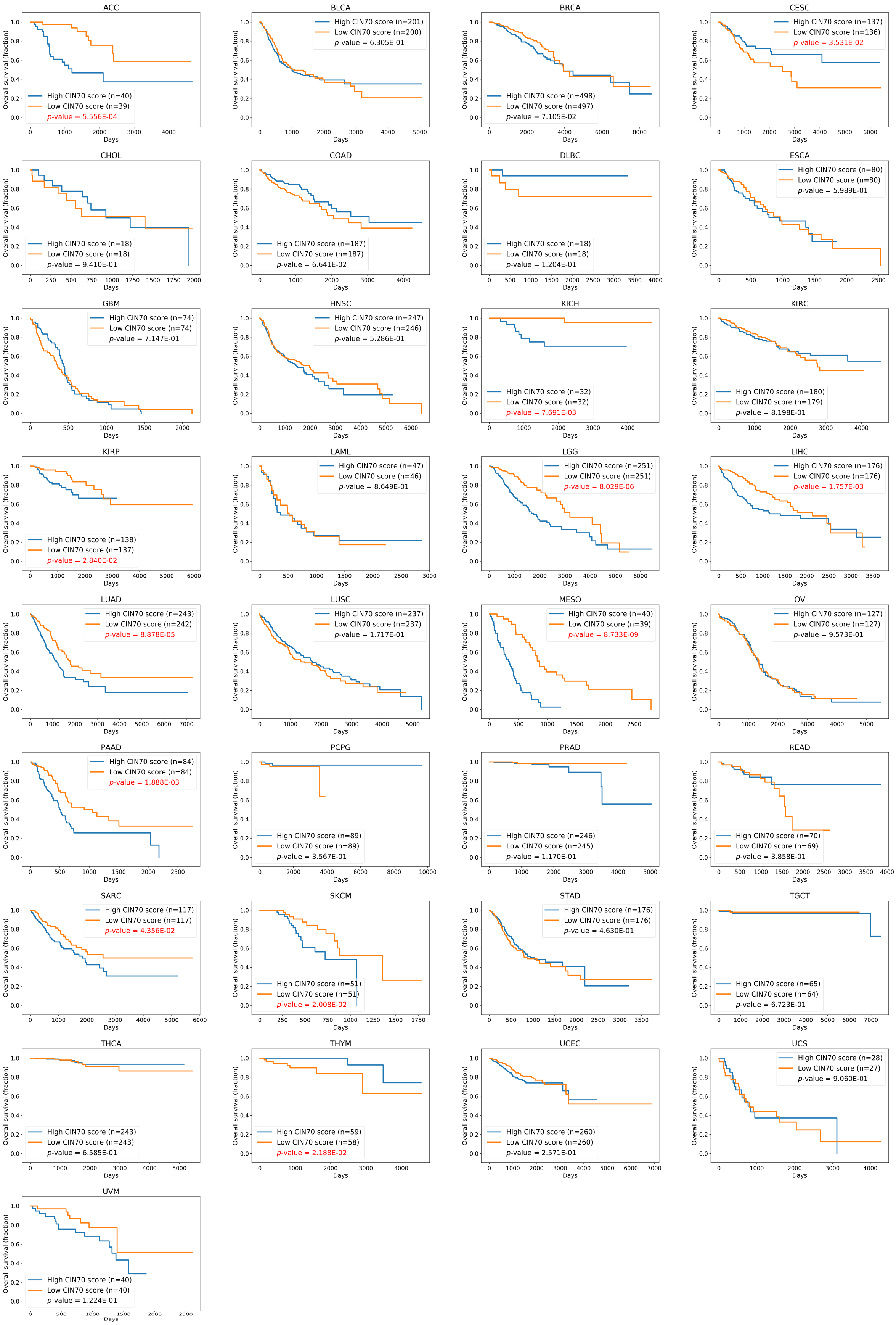

Supplementary Figure S9. Association between CIN70 and overall survival. High/low CIN70 groups are classified according to the median of CIN70 in each cancer type. *p*-values are given based on log-rank test.
